# Towards a standard sampling methodology on online social networks: collecting global trends on Twitter

**DOI:** 10.1007/s41109-016-0004-1

**Published:** 2016-06-01

**Authors:** C. A. Piña-García, Carlos Gershenson, J. Mario Siqueiros-García

**Affiliations:** 1 Instituto de Investigaciones en Matemáticas Aplicadas y en Sistemas, Departamento de Ciencias de la Computación, Universidad Nacional Autónoma de México, Ciudad de México, México; 2 Centro de Ciencias de la Complejidad, Universidad Nacional Autónoma de México, Circuito Maestro Mario de la Cueva S/N, Ciudad Universitaria, Ciudad de México, 04510 México; 3 SENSEable City Lab, Massachusetts Institute of Technology, 77 Massachusetts Avenue, Cambridge, 02139 USA; 4 MoBS Lab, Network Science Institute, Northeastern University, 360 Huntington av 1010-177, Boston, 02115 USA; 5 ITMO University, Birzhevaya liniya 4, St. Petersburg, 199034 Russia

**Keywords:** Twitter, Sampling method, Random walks, Online social network, Data acquisition

## Abstract

One of the most significant current challenges in large-scale online social networks, is to establish a concise and coherent method aimed to collect and summarize data. Sampling the content of an Online Social Network (OSN) plays an important role as a knowledge discovery tool.

It is becoming increasingly difficult to ignore the fact that current sampling methods must cope with a lack of a full sampling frame i.e., there is an imposed condition determined by a limited data access. In addition, another key aspect to take into account is the huge amount of data generated by users of social networking services such as Twitter, which is perhaps the most influential microblogging service producing approximately 500 million tweets per day. In this context, due to the size of Twitter, which is problematic to be measured, the analysis of the entire network is infeasible and sampling is unavoidable.

In addition, we strongly believe that there is a clear need to develop a new methodology to collect information on social networks (social mining). In this regard, we think that this paper introduces a set of random strategies that could be considered as a reliable alternative to gather global trends on Twitter. It is important to note that this research pretends to show some initial ideas in how convenient are random walks to extract information or global trends.

The main purpose of this study, is to propose a suitable methodology to carry out an efficient collecting process via three random strategies: Brownian, Illusion and Reservoir. These random strategies will be applied through a Metropolis-Hastings Random Walk (MHRW). We show that interesting insights can be obtained by sampling emerging global trends on Twitter. The study also offers some important insights providing descriptive statistics and graphical description from the preliminary experiments.

## Introduction

In recent years, there has been an increasing interest in Online Social Networks (OSNs) exploration. Mining social signals can provide quick knowledge of a real-world event (Roy and Zeng 2014). More recently, areas of social network analysis are now expanding to different disciplines, not only in data mining studies but also in computational social science (user behavior), social media analytics and complex systems. Thus, the availability of unprecedented amounts of data about human interactions from different social networks opens the possibility of using this information to leverage knowledge about the diversity of social behavior and the activity of individuals (Lu and Brelsford 2014; Piña-García and Gu 2013b; 2015; Thapen and Ghanem 2013; Weng et al. 2012). The focus of social data analysis is essentially the content that is being produced by users. The data produced in social networks are rich, diverse and abundant, which makes them a relevant source for data science (Ferrara et al. 2014; Kurka et al. 2015; Weikum et al. 2011).

The main challenge faced by many experiments in data science is the lack of a standard methodology to collect and analyze data sets. Thus, the main obstacles that data scientists face is as follows:

1. They do not know what sort of data they need,
2. They do not know how much data they need,
3. They do not know what critical questions they should be asking and
4. They do not know is this data is private i.e. this data could be considered illegal in some contexts.

Social media platforms have increasingly replaced other means of communication, such as telephone and emails (Phan and Airoldi 2015). Thus, the rising interest in digital media and social interactions mediated by online technologies is boosting the research outputs in an emerging field that is multidisciplinary by nature: it brings together computer scientists, sociologists, physicists, and researchers from a wide range of other disciplines (González-Bailón et al. 2014).

Twitter, can be considered the most studied OSN (Kurka et al. 2015). This social media platform provides an efficient and effective communication medium for one-on-one interactions and broadcast calls (e.g., for assistance or dissemination and access to useful information) (Phan and Airoldi 2015). In this regard, we consider Twitter as a a suitable large-scale social network to be explored (Kwak et al. 2010).

Twitter is the most famous microblogging website in the social media space, where users post messages that are limited to 140 characters. In addition, users can follow other accounts they find interesting. Posts are called “tweets”. Unlike the case with other social networks, the relationship does not have to be mutual (Golbeck 2013). It should be noted that Twitter produces approximately 500 million tweets per day, with 271 million regular users (Serfass and Sherman 2015). Therefore, Twitter has been a valuable tool to track and identify patterns of mobility and activity, especially using geolocated tweets. Geolocated tweets typically use the Global Positioning System (GPS) tracking capability installed on mobile devices when enabled by the user to give his or her precise location (latitude and longitude).

In this research we adopt a strategy to identify “trending topics” generated in real-time on Twitter (Weng and Menczer 2015). The main goal of this research is to extract emergent topics and identify their relevance on Twitter. This manuscript is an exploratory data analysis based on topical interest. It is important to note that Twitter has emerged as an important platform to observe relatively informal communication.

This manuscript provides the basic steps that were followed to collect systematically a set of trending topics. These topics or global trends^1^ were gathered and filtered according to their geographic distribution and topical interest.

In addition, we present a statistical and a descriptive analysis of the main features obtained from our collected dataset. Finally, our central hypothesis in this work is that in order to advance our understanding of social interaction, it is necessary to propose a reliable methodology to collect, analyze and visualize collected data from OSNs.

### Contributions

A central hypothesis in this study is that in order to advance our quantitative understanding of social interaction, is not possible to get by with incomplete data. It becomes necessary to obtain representative data. Therefore, the aim of this study is to propose an algorithm to discover and collect emerging global trends on Twitter. Specifically, our contributions in this study are as follows:

- This paper provides a series of random strategies (Brownian, Illusion and Reservoir) based on random walk models to sample small but relevant parts of information produced on Twitter.
- This research is intended to determine the extent to which random walks can be combined by using an alternative version of a Metropolis-Hastings algorithm.

## Related work

A considerable amount of literature has been published on using graph sampling techniques on large-scale OSNs. These studies are rapidly growing in the scientific community, showing that sampling methods are essential for practical estimation of OSN properties. These properties include, for example: user age distribution, net activity, net connectivity and node degree. Studies on social science show the importance of graph sampling techniques, e.g., (Caci et al. 2012; Fire and Puzis 2012; Lee et al. 2006; Mislove et al. 2006; Scott 2011).

Online social networks such as Facebook represents one of the biggest social services in the world. Therefore, it may be seen as a large-scale source to collect data with the aim to obtain a representative sample or characterize the whole network structure (Bhattacharyya et al. 2011; Caci et al. 2011; Ferri et al. 2012; Ugander et al. 2011). Recent evidence suggests that efficient random walk inspired techniques has been successfully used to sample large-scale social networks, in particular, Facebook (Gjoka et al. 2010; 2011a). However, despite its relative success of Facebook, these specific sampling strategies have not been tested on different social networking services such as: Twitter.

A number of researchers have pointed out that statistical approaches such as random walks can be used to improve and speeding up the process of sampling. This can be done by considering different randomized algorithms which are able to cope with large datasets. Recently, the Metropolis-Hastings Random Walk algorithm have been tested on Facebook and Last.fm (a music website with 30 million active users) showing significant results for an unbiased sampling of users (Gjoka et al. 2011a, 2011b; Kurant et al. 2011).

Similarly, some studies based on supervised random walks use the information from the network structure with the aim to guide a random walk on the graph, e.g., on the Facebook social graph (Backstrom and Leskovec 2011). In addition, there are other studies that introduce the same random walk technique analyzed from the Markov Chain Monte Carlo (MCMC) perspective, i.e., the Metropolis-Hastings random walk (MHRW), which is mainly used to produce uniform samples (Bar-Yossef and Gurevich 2008).

An alternative Metropolis-Hastings random walk using a spiral proposal distribution is presented in (Piña-García and Gu 2013b). The authors examined whether it was possible to alter the behavior of the MHRW using spirals as a probability distribution instead a classic *Gaussian* distribution. They observed that the spiral inspired approach was able to adapt itself correctly to a Metropolis-Hastings random walk.

These studies presented thus far provide evidence that there is a growing interest in the use of rapid sampling models and a clear need of data extraction tools on Facebook (Bhattacharyya et al. 2011; Caci et al. 2011; Ferri et al. 2012; Ugander et al. 2011). However, Twitter has recently received special attention from researchers that are interested in uncovering global topics, that are well known as: “memes” and “hashtags^2^” (Hawelka et al. 2014; Kallus 2014; Mitchell et al. 2013; Takhteyev et al. 2012; Thapen and Ghanem 2013).

Recently, there has been an increasing amount of literature on data collection via Twitter. Preliminary work on information diffusion was presented in (Weng et al. 2013b), where authors examined the mechanisms behind human interactions through an unprecedented amount of data (social observatory). They also argued that information diffusion affects network evolution.

An important analysis about the geography of twitter networks was presented in (Takhteyev et al. 2012). In this case, the authors showed that distance matters on Twitter, both at short and longer ranges. In addition, they argued that the distance considerably constrains ties. The authors highlighted the importance of Twitter in terms of collection of data due to its popularity and international reach. They also suggested that these ties at distances of up to 1000 km are more frequent than what it would be expected if the ties were formed randomly.

In a large longitudinal study carried out in (Hawelka et al. 2014), the authors found global patterns of human mobility based on data extracted from Twitter. A dataset of almost a billion of tweets recorded in 2012, was used to estimate volumes of international travelers. The authors argue that Twitter is a viable source to understand and quantify global mobility patterns.

Furthermore, a detailed investigation on correlations between real-time expressions of individuals and a wide range of emotional, geographic, demographic and health characteristics was conducted in (Mitchell et al. 2013). Results showed how social media may potentially be used to estimate real-time levels and changes in population-level measures (Haralabopoulos and Anagnostopoulos 2014; Leskovec et al. 2008). The findings in (Mitchell et al. 2013), were supported by a large dataset of over 10 million geo-tagged tweets, gathered from 373 urban areas in the United States during the calendar year of 2011.

In another major study, a “conversational vibrancy” framework to capture dynamics of hashtags based on their topicality, interactivity, diversity, and prominence was introduced in (Lin et al. 2013). The authors examined the growth and persistence of hashtags during the 2012 U.S. presidential debates. They point out that the growth of a hashtag and death is largely determined by an environmental context condition rather than the conversational vibrancy of the hashtag itself.

A recent study about community structure in OSNs was presented in (Weng et al. 2013a). Authors claim that “memes” and behaviors can be mimicked as a contagion phenomenon. The authors concluded that the future popularity of a meme can be predicted by quantifying its early spread patterns.

A very important study that builds a systematic framework for investigating human behaviors under extreme events with online social network data extracted from Twitter was carried out in (Lu and Brelsford 2014). The researchers have shown distinctive changes in patterns of interactions in online communities that have been affected by a natural disaster compared to communities that were not affected.

Finally, in a controlled study of the automatic analysis of UK political tweets was provided in (Thapen and Ghanem 2013). In this case, the authors examined the extent of which the volume and sentiment of tweets can be used as a proxy to obtain their voting intentions and compare the results against existing poll data. In addition, the authors propose a data collection method through a list of selected Twitter accounts classified by party affiliation. Approximately 689,637 tweets, were retrieved from the publicly available timelines of the members of Parliament on 10th June 2013, the authors took a random sample of 600 users from Twitter.

## Problem definition

As the digital world grows, it generates an enormous amounts of data every second, challenging us to find new methods to efficiently extract and sample information. Currently, data science is a relatively new field of study that involves concepts that come from data analysis and data mining. However, we are experiencing a digital revolution where collecting data has become in a everyday task to data scientist. In this regard, scientific production based on data science has grown up sharply in last few years and it tends to still happen. At this point, it is possible to observe many proposed methodologies to collect and analyze information. However, none of these approaches provide us a truthful unified framework in data science. Thus, it becomes necessary to propose a standard sampling methodology that allows us to establish a general framework to cope with current challenges.

Although extensive research has been carried out on related work about data collection, much uncertainty still exists about the existence of a standard sampling methodology to efficiently collect datasets from social networking services. Many of the related work aforementioned have based their experiments on offline datasets i.e., this information have been previously collected by third parties without revealing in some cases how the data was obtained from the source. In addition, the previous studies lack of a standardized random sampling model, which means that each study applies different techniques for sampling OSNs.

Recent evidence suggests that an uniform sample can be obtained with a remarkable performance in terms of low computational cost (Gjoka et al. 2010; 2011a). It is important to note that our approach is mainly based on this work carried out by Minas Gjoka et al. Further information and material can be found on http://www.minasgjoka.com/index.html.

The key research question of this study is whether or not is possible to use a different approach, apart from the normal or Gaussian distribution as an internal random generator. Thus, this paper seeks to exploit ideas from randomized algorithms such as: a Brownian walk (based on a normal distribution), a spiral-inspired walk and a Reservoir sampling algorithm.

However, in this manuscript we are focused in extracting global trending topics as a case of study. It is important to mention that since at the moment there is not a clear solution or methodology in terms of data extraction i.e., there is not any “ground truth” that we can follow or compare with. This proposed methodology pretends to show some initial ideas in how convenient are random walks to extract information or trends in this particular case.

## Random strategies

This section will examine three random strategies that are incorporated into the alternative version of the MHRW. These random strategies are aimed to be used heuristically as an internal picker for a candidate node (hereinafter referred to as *ϱ*). This set of random strategies is composed as follows: *Brownian* walk (normal distribution), a spiral-inspired walk (Illusion) and a Reservoir sampling method. It is important to note that the Brownian case will be used as a the baseline to be compared with the rest of the random strategies.

The main idea of the Metropolis-Hastings algorithm is to provide a number of random samples from a given distribution. Thus, our proposed version of the MHRW is able to sample a candidate node *ϱ*, which is directly obtained from: *q*(*y*|*x*)={*B* *r* *o* *w* *n* *i* *a* *n*,*I* *l* *l* *u* *s* *i* *o* *n*,*R* *e* *s* *e* *r* *v* *o* *i* *r*}.

### Brownian walk

The traditional approach used to sample through the MHRW is based on the normal distribution. In this regard, we have developed a *Brownian* walk that presents a normal distribution. It is important to note that in most cases the Brownian walk is related to a continuous time process. However, in this research it has been considered a discretized version of this strategy. Technically speaking in this model, the candidate node *ϱ* will be computed according to the Java language command: Math.random().

### Illusion spiral

**Fig. 1:**
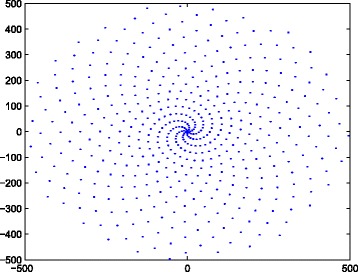
Pattern visualization of the *Illusion* spiral

In this research, we have considered a spiral-inspired approach in terms of an *Illusion* spiral.^3^ This spiral presents an interesting geometric shape which presents a sequence of points spirally on a plane such that they are equitably and economically spaced (see Fig. 1). This spiral model is produced by the following expression:

1 $$ z \leftarrow az + bz/\left|z\right| \quad.  $$

Where *a*=0.6+0.8*i* and *b*=0.65+0.7599*i*. It is important to note that this spiral-inspired approach should not be considered as a formal distribution in itself. It is just a consequence of making use of complex numbers to correctly generate a geometric shape or in this case an Illusion spiral. In addition, Eq. 1 includes complex variables to correctly generate the pattern.

Thus, a *z* value ($z \in \mathbb {C}$) of the form *z*=*a*+*b* *i* is iteratively generated. In this regard, we are able to obtain a collection of complex numbers where the real part will be considered as a candidate node *z*=*ϱ* for our study purposes.

### Reservoir sampling

A reservoir sampling can be seen as an algorithm that consists in selecting a random sample of size *n*, from a file containing *N* records, in which the value of *N* is not known to the algorithm. According to (Vitter 1985), the first step of any reservoir algorithm is to put the first *n* records into a “reservoir”. The rest of the records are processed sequentially. Thus, the number of items to select (*k*) is smaller than the size of the source array *S*(*i*). Algorithm 1 provides an overview of the steps carried out by the reservoir sampling process.



In this study, a non-conditional version of the aforementioned algorithm will be considered i.e., lines 8–10 are discarded. In this case, the random number *j*:=*r* *a* *n* *d* *o* *m*(1,*i*) has come to be used to refer to a candidate node *j*=*ϱ*.

## The alternative version of the metropolis-hastings algorithm

The Metropolis-Hastings algorithm makes use of a proposal density: *q*(*y*|*x*) which might be a simple distribution such as normal. For this study purposes, the term *q*(*y*|*x*) will be applied to a set of three mutually exclusive random strategies: *q*(*y*|*x*)={*B* *r* *o* *w* *n* *i* *a* *n*,*I* *l* *l* *u* *s* *i* *o* *n*,*R* *e* *s* *e* *r* *v* *o* *i* *r*}.

**Fig. 2:**
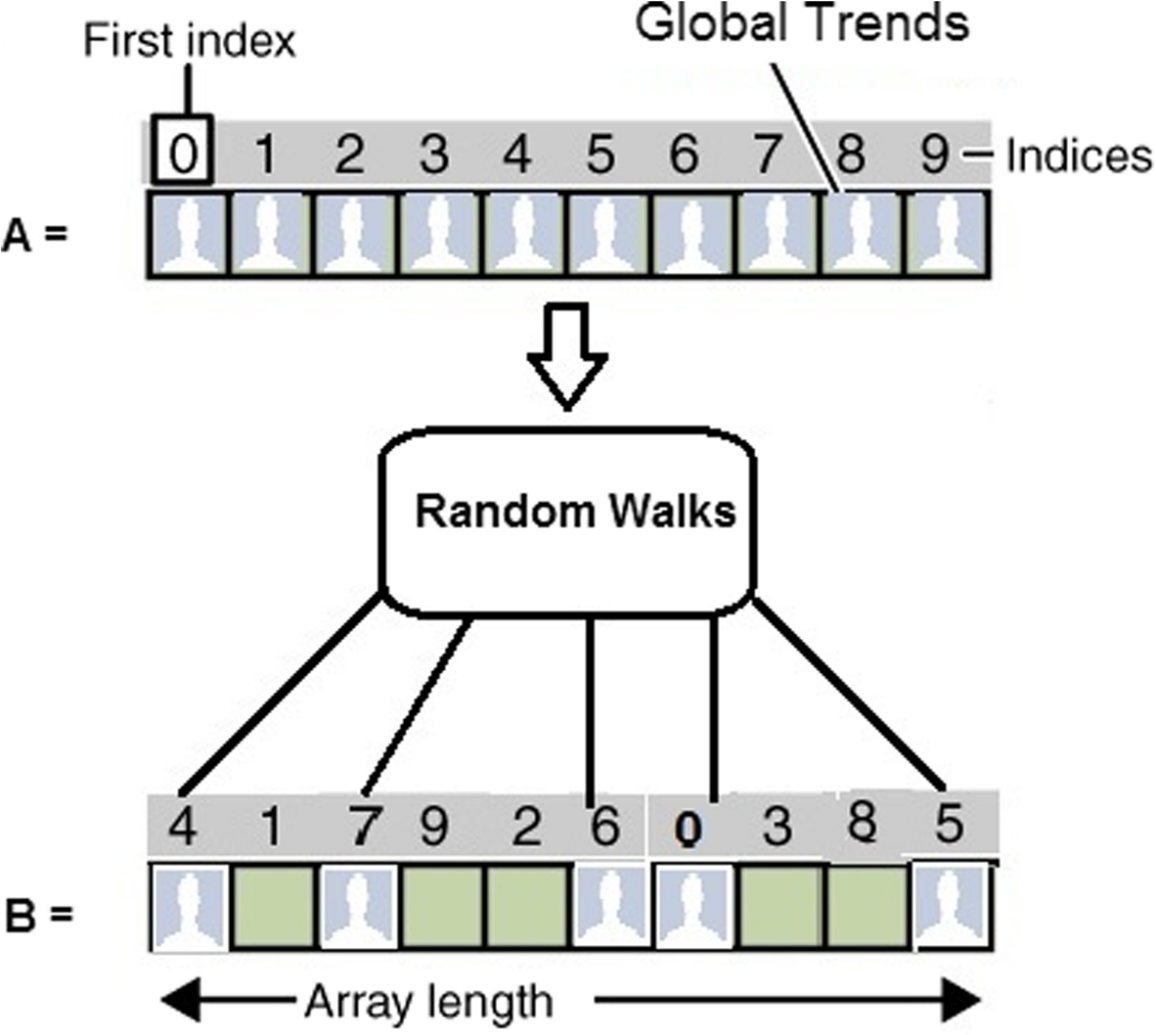
In summary, all the global trends retrieved from Twitter are potential nodes. Subsequently, all the trends that were collected from the servers are drawn through the random walks provided by *q*(*y*|*x*)

In this context we conceive a random strategy as a procedure designed to generate a sequence of random numbers, this sequence represents global trends on Twitter. One of the three random strategies is selected to pick different trends from list **A**. Subsequently, all the trends that were sampled are copied to a matrix **B**, these collected trends are arranged according to how they were chosen (see Fig. 2).

The key idea of this alternative version of the MHRW algorithm is to generate a number of independent samples from a given random generator. Thus, it is necessary to sample a candidate node *ϱ* from *q*(*y*|*x*)={*B* *r* *o* *w* *n* *i* *a* *n*,*I* *l* *l* *u* *s* *i* *o* *n*,*R* *e* *s* *e* *r* *v* *o* *i* *r*}. The candidate node is accepted if and only if this node belongs to the graph *G*. The steps of this method are outlined in Algorithm 2.


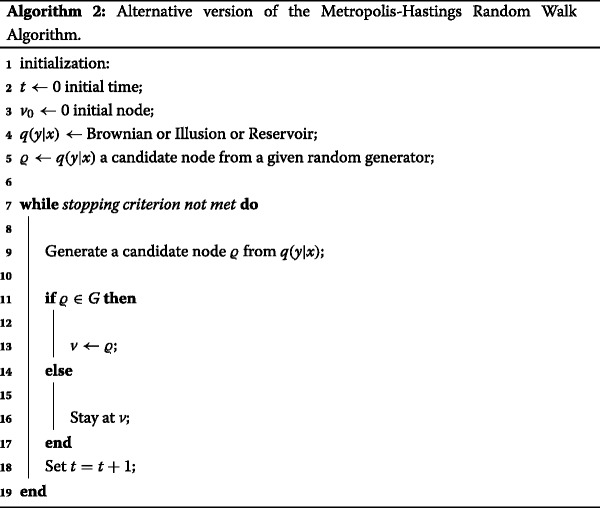


It should be highlighted that the node *v*_0_ is placed in terms of the first record retrieved from the servers of Twitter. Similarly, the stopping criterion is determined by the number of countries randomly chosen e.g., it is possible to select 15 countries with their respective top 10 trending topics (15 × 10 trends in total). The next section will describe how these countries are obtained.

## Sampling global trends on Twitter

### Pre-processing

**Fig. 3:**
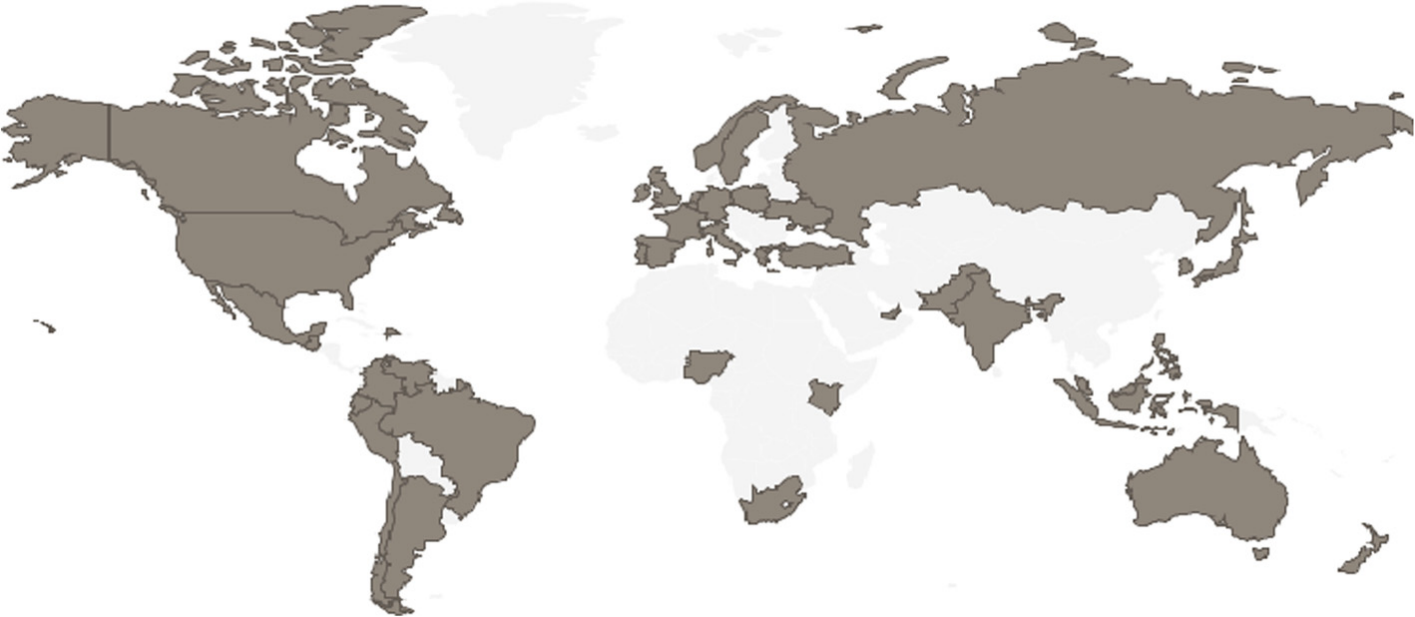
The map shows the geographical location of the countries around the globe that had more activity on Twitter according to a set of empirical trials

For the estimation of trends concentration, a list of countries with publicly available trends was requested from Twitter. Countries are identified by means of a specific WOEID. The term WOEID refers to a service that allows to look up the unique identifier called the “Where on Earth ID” (see http://developer.yahoo.com/geo/geoplanet/). Figure 3 illustrates a map of the geographical locations of these countries. In addition, a full list of retrieved countries can be found in Table 1.

**Table 1:** Table of retrieved countries with more activity on Twitter during our empirical trials

List of countries	
Argentina	Australia
Belgium	Brazil
Canada	Chile
Colombia	Dom. Republic
Ecuador	France
Germany	Greece
Guatemala	India
Indonesia	Ireland
Italy	Japan
Kenya	Korea
Malaysia	Mexico
Netherlands	New Zealand
Nigeria	Norway
Pakistan	Peru
Philippines	Poland
Portugal	Russia
Singapore	South Africa
Spain	Sweden
U. Arab Emirates	Turkey
Ukraine	United Kingdom
United States	Venezuela

**Fig. 4:**
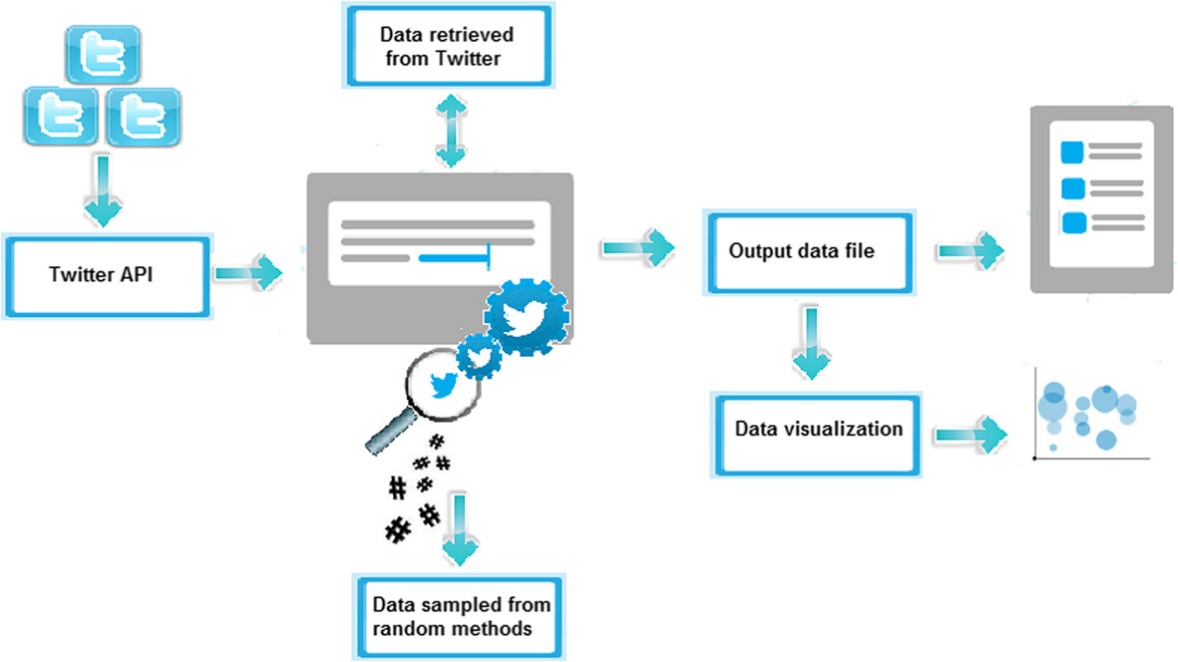
The diagram shows the content extraction tool or social explorer using an API to establish a connection, then a random sampling is carried out for collecting global trends in real-time from Twitter. Finally, it generates an output information file and depicts the results upon the visual interface

The Algorithm 2 interacts with Twitter via its public API as a primary way to retrieve data. Once all the information has been retrieved, a random sampling is performed across the global trends using Algorithm 2. Collected samples are stored in an output data file and depicted on a visual interface. Figure 4 shows how this process works on Twitter.

A sample is chosen according to the following eligibility criteria (initial conditions): 1) number of countries and 2) a minimum number of users following a global trend. In this study, the initial conditions consisted of 15 countries and 10 users. Therefore, a maximum of 150 (15 × 10) trending topics per independent run were available to be gathered. However, due to a trending topic or an user can be counted multiple times, which makes the measurement hard to interpret, all duplicate trends and duplicate users were removed from the sample. After filtering out all duplicates, it can be built a data structure containing a set of unique records.

In summary, the steps to generate the data are as follows:

1. Collect a list of WOEIDs by searching countries with publicly available trends, then select randomly a set of *W*=15 unique countries.
2. for each country *c*∈*W*, we acquire a list of the top ten trending topics(*TT*), add each trending topic *TT* as a node to the graph *G*. Then, set the minimum number of users following this trend to *F* *r*=10;
3. for each *TT*, get a list of users linked to the corresponding trending topic, e.g. *F* *r*(*T* *T*), and add each of them as a node to *G*;
4. create an edge [*T* *T*,*F* *r*(*T* *T*)] and add it to *G*;
5. save the graph *G*.

## Results

In order to assess the performance of the social explorer, a sample of publicly available trends was collected, this random sample contains tweets posted from December 17 to December 20 2013, between 16:30 and 22:30 GMT (time window). This sample consisted of 3,325 trending topics generated by 225,102 unique users that emerged during the observed time window.

It is important to note that in this case, not only tweets written in English were extracted. This feature provides a different framework with respect to previous studies whereby only English tweets were collected e.g., (Weng et al. 2012, 2013a). One advantage of this multilingual feature is that it avoids a bias in terms of the information posted in English.

To replicate the sampling process, a series of 10 independent walks was performed for each one of the three random strategies: *q*(*y*|*x*)=Brownian, Illusion, Reservoir (30 runs in total). Then, two different output files were stored for further analysis: a .dat file and a .gml file. The first one contains information such as: Total number of trending topics, total number of unique followers, number of iterations, total number of sampled trends, a full list of the collected trends, number of nodes, number of edges, node degree per trending topic, memory usage, total number of duplicates and the elapsed time during the sampling process. On the other hand, the second output file contains a GML (Graph Modeling Language) formatted file, which describes a graph obtained by the social explorer. This file is used to build and evaluate graphically each one of the samples.

**Fig. 5:**
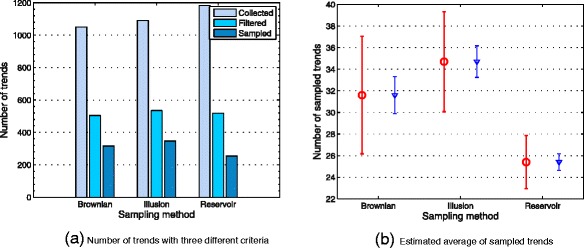
**a** Plot divided into three main criteria: number of trends retrieved from Twitter (*collected*); number of trends after removing all duplicates (*filtered*) and number of sampled trends collected by each random generator (*sampled*). **b** Means corresponding to the average of sampled trends

Figure 5 a compares a cumulative analysis of the number of trends. This plot may be divided into three main criteria: number of trends retrieved from the Twitter service (collected), number of trends after removing all duplicates (filtered) and number of sampled trends collected by each random generator (sampled). Similarly, means with respect to the number of sampled trends are shown in Fig. 5 b. What is interesting in this data, is that the sampled trends represents the core information to evaluate how was the three models behavior in terms of data collection. It should be highlighted that the number of collected trends depends exclusively from the Twitter service. Likewise, the filtering process was carried out as a cleaning data process.

**Fig. 6:**
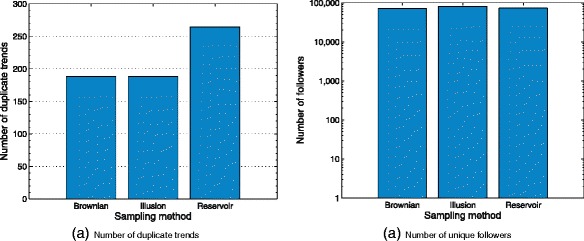
Plots generated during the observation time window: **a** the number of duplicate trends for *q*(*y*|*x*). **b** the number of unique followers presented with logarithmic scale for the y-axis

Owing to the natural tendency of the social explorer to move toward the same node many times, which is induced by each one of the random strategies, a considerable number of duplicate trends is added to the output sequence. This permits to compare the results in terms of the number of duplicate trends generated during the observation time window (see Fig. 6 a). Likewise, Fig. 6 b compares the number of unique followers obtained from each random generator *q*(*y*|*x*).

Table 2 compares a descriptive statistics of the average number in terms of: trends filtered, sampled trends, duplicate trends and the number of followers. It is apparent from this table that the Illusion model slightly differs from the rest of the random strategies in terms of sampled trends. This difference may be caused by the spread-out pattern presented on the shape of this spiral (see Fig. 1).

**Table 2:** Descriptive statistics during the observation time window

Trends	*q*(*y*|*x*)	Total	Avg	Std
Filtered	Brownian	504	50.4	8.16
	Illusion	535	53.5	7.59
	Reservoir	518	51.8	4.07
Sampled	Brownian	316	31.60	5.44
	Illusion	347	34.70	4.62
	Reservoir	254	25.40	2.45
Duplicated	Brownian	188	18.80	3.08
	Illusion	188	18.80	3.04
	Reservoir	264	26.40	2.91
Followers	Brownian	71574	7157.4	1696.6
	Illusion	80522	8052.2	2654.8
	Reservoir	73006	7300.6	1582

**Fig. 7:**
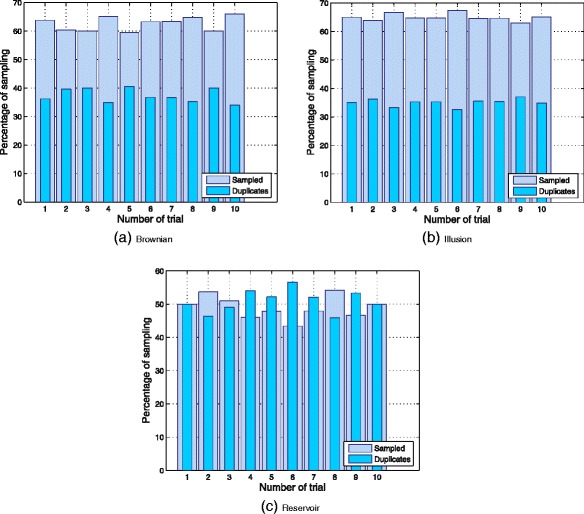
Group of plots of the percentage of accuracy plotted versus the number of trials. The measures are computed based on the percentage of sampled trends and on the percentage of duplicate trends generated by each random generator

In addition, the percentage of accuracy was computed based on the ratio obtained by dividing the cumulated value of sampled trends by the total number of iterations carried out by the social explorer. The same procedure was applied to the cumulated value of duplicate trends. The results obtained from this analysis are summarized in Fig. 7. This set of plots considers the percentage amounts of sampled and duplicate trends over all the 30 samples produced by each random generator. Data from this figure can be compared with the data in Table 3 which shows that the Brownian and Illusion models perform well during their 10 independent runs. However, the reservoir model showed a poor performance in terms of percentage of sampling.

**Table 3:** Descriptive Statistics of the average amount of sampled and duplicate trends. It can be seen from the data that the Illusion spiral outperforms all others random strategies in terms of the number of sampled trends

Trends	*q*(*y*|*x*)	Avg
% Sampled	Brownian	62.61 %
	Illusion	64.94 %
	Reservoir	49.06 %
% Duplicated	Brownian	37.3 %
	Illusion	35.05 %
	Reservoir	50.93 %

Basic statistics of the average amount of the relative accuracy in sampling and the average amount of the percentage of duplicate trends, are reported in Table 3.

### Memory consumption

This section examines the estimated memory usage employed by each proposed model. The results obtained from the preliminary analysis of memory consumption can be compared in Table 4, this table compares the average memory consumption in Megabytes (MB) and the total of memory used across 10 independent runs. In this regard, there were no significant differences between the amount of MB used for each random generator.

**Table 4:** Descriptive Statistics of memory consumption. This table compares the average memory consumption in Megabytes (MB) and the total of memory used across 10 independent runs

Results	*q*(*y*|*x*)	Total	Avg	Std
Memory (MB)	Brownian	66	6.6	4.16
	Illusion	66	6.6	2.71
	Reservoir	62	6.2	4.61

**Fig. 8:**
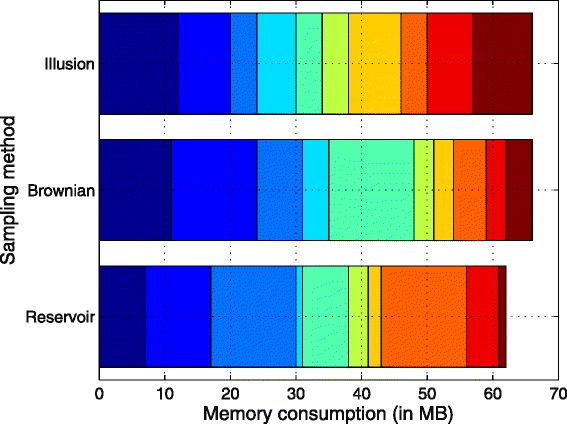
Stacked bar chart displaying the sum of the memory consumption split in 10 independent runs per random generator

Due to the experiments were run using custom software written in Java, it has been considered to assess the results obtained related to the memory consumption in Megabytes (MB). The basic computer hardware information is as follows: Processor: Intel(R) Core(TM)2 Duo CPU at 3.33 GHz. Installed memory (RAM): 4.00 GB. System type: 64-bit operation system. The application was run on Windows 7 Enterprise edition. Figure 8 presents a cumulative memory usage plot. This plot is presented as a stacked bar which provides the sum of all the memory consumption across 10 independent walks per model. From these data, it can be seen that there were no significant differences between the sampling methods used as random strategies.

### Concentration levels of trending topics

In order to identify the concentration levels of the trending topics obtained from the .gml file, three samples were used to analyze visually the distribution of the clusters. It is important to note that these samples were chosen because they have a greater content of trending topics than the rest of the samples, each sample corresponds to one random generator. Thus, data was plotted as a community structure.

**Fig. 9:**
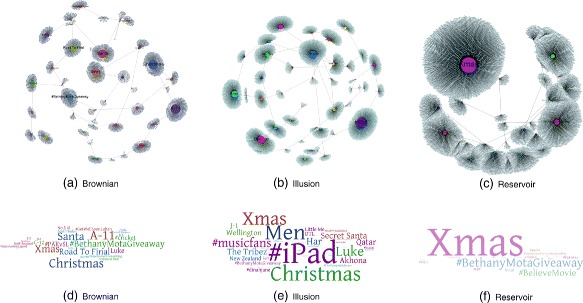
Visualizations of concentration levels of trending topics for the following random strategies: **a** Brownian, **b** Illusion and (**c**) Reservoir. These graphs represent trending topics produced by Twitter users over our sampling time window. The size of a node indicates the degree of a trend. Similarly, word clouds in (**d**) Brownian, **e** Illusion and (**f**) Reservoir show a group of words whose sizes are proportional to the number of edges incident to the trending node i.e., the degree of the node. Essentially, either the graphs and the word clouds show the same information

As can be seen from the graphs in Fig. 9 a, b and c the number of edges incident to the node defines the level of dominance of each trending topic. From the chart, it can be seen that by far the greatest trending topic used by the users of Twitter is related to Christmas season e.g., “Xmas”, “Christmas” and “Santa”. The most likely cause of this outcome is due to the fact that the time window of the experiments was in December. Similarly, it can be seen from the *word clouds* in Fig. 9 d, e and f the same information about the concentration levels of the trending topics. In this case, the size of a word is proportional to the relative degree of a trend. At this stage, it is possible to distinguish more words than the graph version depicted in figures: Fig. 9 a, b and c. Essentially, either the graphs and the word clouds show the same information.

### Convergence monitoring

Part of the aim of this research is to identify convergence during the sampling process. Therefore, a convergence analysis was prepared according to the procedure used by the Geweke to evaluate the accuracy of sampling-based approaches (Geweke 1991; Lee et al. 2006). This Geweke diagnostics is a standard Z-score which consists in taking two non-overlapping parts of the Markov chain and compares the means of both parts, using a difference of means test to see if the two parts of the chain are from the same distribution (null hypothesis).

This diagnostic represents a test of whether the sample of draws has attained an equilibrium state based on the first 10 *%* of the sample of draws, versus the last 50 *%* of the sample of draws. If the Markov chain of draws has reached an equilibrium state, it would be expected to obtain roughly equal averages from these two splits of the sample (Lesage 1999). MATLAB functions that were used to implement these estimations can be found at http://www.spatial-econometrics.com/gibbs/contents.html.

**Fig. 10:**
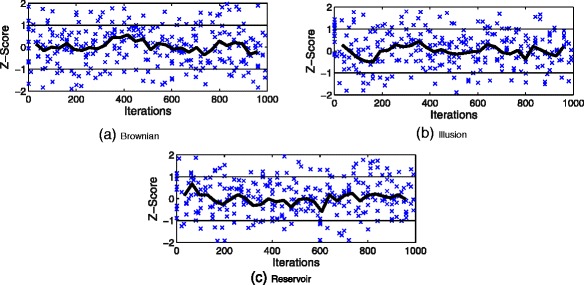
Plots of the resulting Z-scores against the number of iterations for the metric of node degree (number of users that follow a particular trend). *Horizontal lines* at *Z*=±1 are added to the plots to indicate the convergence interval

Figure 10 provides trace plots for the property of node degree (number of users that follow a particular trend). These plots present the Z-score value against the number of iterations. Therefore, using the Geweke diagnostics it is possible to identify the convergence analysis for the Brownian walk, the Illusion spiral and the Reservoir sampling. The number of draws was fixed to 1100 with a *burn-in* process discarding the first 100. Thus, in accordance to (Gjoka et al. 2011a) we can declare convergence when most values fall in the [–1, 1] interval. Additionally, we plot an average line using 30 points on the x-axis. Finally, as it can be seen in Fig. 10 our convergence analysis suggests that our sample draws have attained an equilibrium state showing that the means of the values converge rapidly in the sequence.

## Limitations

One advantage of this approach is the multilingual feature which avoids a bias in terms of the information posted in English. However, there are certain drawbacks associated with the use of different languages e.g., lack of knowledge of the language and the misinterpretation of the statements.

On the other hand, this research does not take into account that the social explorer is not able to distinguish between Twitterbots^4^ and real users on Twitter. Therefore, all the estimates include Twitterbots causing an over estimation in the results. These data must be interpreted with caution since all the information collected from this study is mainly based on the Twitter response service.

## Conclusions

This paper has explained the central importance of defining a standard sampling methodology applicable to cases where the social network information flow is readily available. The main purpose of the current study was to assess a low computational cost method for sampling emerging global trends on Twitter.

It is now possible to state that the development of a faster randomized algorithm able to carry out a collecting process via three random strategies, can be effectively gathered by using *q*(*y*|*x*)={*B* *r* *o* *w* *n* *i* *a* *n*,*I* *l* *l* *u* *s* *i* *o* *n*,*R* *e* *s* *e* *r* *v* *o* *i* *r*}. The present paper confirms previous findings related to the good performance of the Brownian and Illusion generators (Piña-García and Gu 2013a). It should be noted that according to the first systematic study of using a Metropolis-Hastings Random Walk (MHRW) reported by Gjoka et al. (2010), where the authors demonstrated that the MHRW perform well under Facebook using a normal distribution as a random generator (a Brownian walk in this study), the results of this research show that the MHRW, is eligible to be modified in terms of how it heuristically generates a candidate node using different random strategies such as: Illusion and Reservoir.

The empirical findings of this study suggest that, sampling global trends on Twitter has several practical applications related to extract real-time information. Despite its exploratory nature by looking at how impactful people are about a specific topic and within specific categories, this research offers some insight into how to collect publicly available trends using a social explorer, which works as an interface between a faster randomized algorithm proposed in Algorithm 2 and Twitter.

Overall, our current study indicates that our sampling methodology may be a promising new approach to social networking service analysis and an useful exploration tool for social data acquisition. However, a debate continues about the best strategies to follow in this data science context. The controversy about a sampling methodology has raged in last years claiming the need of an standard methodology to collect data on OSNs. No agreement have been achieved within the scientific community in terms of a theoretical framework. Thus, this study highlights the importance of proposing a standard sampling methodology to advance our knowledge for addressing questions of social mining.

## Endnotes

^1^ A word, phrase or topic that is tagged at a greater rate than other tags is said to be a trending topic.

^2^ A hashtag is a word or metadata tag prefixed with the hash symbol (#).

^3^ see (Davis 1993) for a full description of the Illusion spiral.

^4^ A Twitterbot is a program used to produce automated posts on the Twitter microblogging service, or to automatically follow Twitter users.

## Availability of data and materials

Data have been collected through the public Twitter API (https://dev.twitter.com/overview/api). To comply with Twitter terms of service, data cannot be publicly shared. Interested future researchers may reproduce the experiments by following the procedure described in the paper. Anonymized data may be available upon request from Dr. Carlos Piña (carlos.pgarcia@iimas.unam.mx).
